# Conspecific migration and environmental setting determine parasite infracommunities of non-migratory individual fish

**DOI:** 10.1017/S0031182021000780

**Published:** 2021-08

**Authors:** Eloïse C. Rochat, Jakob Brodersen, Isabel Blasco-Costa

**Affiliations:** 1 Natural History Museum of Geneva, Route de Malagnou 1, Geneva 1208, Switzerland; 2 Department of Arctic and Marine Biology, UiT The Arctic University of Norway, Langnes, PO Box 6050, Tromsø 9037, Norway; 3 Department of Fish Ecology and Evolution, EAWAG Swiss Federal Institute of Aquatic Science and Technology, Centre of Ecology, Evolution and Biogeochemistry, Seestrasse 79, Kastanienbaum CH-6047, Switzerland; 4 Department of Aquatic Ecology & Evolution, Institute of Ecology and Evolution, University of Bern, Bern 3012, Switzerland

**Keywords:** Groundwater, non-metrical multidimensional scaling, parasite infracommunity, partial migration, surface-water

## Abstract

Parasite infracommunities tend to be stochastic in nature, although environmental characteristics such as the type of water source in streams and host traits can have an effect on the biotic assemblages and by extension the parasite fauna. We examined the effect of water source and the rate of adult fish migration on the metazoan parasite infracommunities of conspecific juvenile brown trout, *Salmo trutta* L. among streams flowing into Lake Lucerne (Switzerland). Juvenile (1 to 2-year old) fish harboured higher parasite species richness in groundwater-fed than in surface water-fed streams, whereas the rate of fish migration did not affect infracommunity richness. Heteroxenous species were more common in groundwater-fed streams with high and medium rates of trout migration, whereas infracommunities in surface water-fed streams and streams with low rates of fish migration were dominated by one monoxenous parasite or lacked infections. Similarity in the parasite infracommunity composition of juvenile trout across streams was explained by the interaction between type of water source and adult migration rates. Our conclusions support that similarity in the parasite composition of resident freshwater conspecifics can be predicted by the local environmental settings and host migratory behaviour, whereas parasite richness is mainly influenced by the environmental characteristics.

## Graphical abstract

**Figure:**
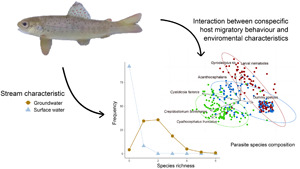

## Introduction

Parasite communities and species distributions are influenced by multiple abiotic and biotic variables that lead to their non-random distribution in space (Crofton, 1971; Ostfeld *et al*., 2005). Among the biotic factors, host vagility and behaviour can influence the dispersal of parasites and their distribution. For instance, a migratory host may facilitate the spread of a parasite into new environments where the parasite might encounter and infect a novel host population (e.g. Figuerola and Green, 2000; Altizer *et al*., 2011; Kutz *et al*., 2013). On the other hand, migration may allow the host to escape parasitism by moving away from infection hotspots (e.g. Bartel *et al*., 2011; Poulin *et al*., 2012) and reduce the exposure of juveniles to parasitism by removing infected adult individuals from the area (e.g. Krkošek *et al*., 2007). A link between host migration and similarity in the parasite communities has been extensively documented in the literature. Parasites have often been used to trace the migratory behaviour of their hosts and discriminate host stocks in the sea, but rarely in freshwater (e.g. Carballo *et al*., 2012; Sheehan *et al*., 2016; Irigoitia *et al*., 2017; Canel *et al*., 2019). However, migration is a heterogeneous phenomenon, varying both between and within species. Partial migration is common amongst migratory species, particularly in fish, where just a fraction of a population migrates and the remainder stay resident (Chapman *et al*., 2012). Whereas previous work has focussed on how migratory individuals within migratory populations are affected by parasitism, we know much less about how the migratory life style of some individuals may influence parasite infections of non-migratory individuals of the same populations.

Parasite infracommunities of freshwater fish are generally considered stochastic and unpredictable in nature, and represent an independent assortment of parasites assembled from a pool of regionally available species (e.g. Kennedy *et al*., 1978; Esch *et al*., 1988; Kennedy, 1990; Guégan *et al*., 2005). Abiotic factors at the local scale can act as filters of the pool of organisms found at higher scales (Poff, 1997). For instance, the physical and chemical properties of the water can vary substantially depending on the source of the water in the stream and lead to changes in the free-living and parasitic faunal communities (Ward, 1994). In surface-water-fed streams, water originates mostly from snow melting and rain in the mountains. These streams represent variable environments with low nutrients in the water and little vegetation. These factors lead to a poorer macroinvertebrate community and lower abundance of species than in groundwater-fed streams (Ward, 1994). The latter have a large content of nutrients and elements washed from the substrate, like nitrogen, and support more diverse communities of species than surface water streams. In general, the richer and more abundant the macroinvertebrate community, the more likely it is to support diverse parasite species (e.g. Kennedy *et al*., 1986; Sures, 2004; Johnson and Heard, 2017), in particular heteroxenous ones that require multiple host to complete a single generation (e.g. Marcogliese, 1995; Hernandez *et al*., 2007). Abiotic processes like the unidirectional water flow in streams can structure free-living and parasite populations and communities by favouring a downstream dispersion of small organisms, thus increasing their concentration downstream (Blasco-Costa *et al*., 2013; Paterson *et al*., 2018; Tonkin *et al*., 2018). Furthermore, the environmental conditions of streams may affect both phenotype and foraging ecology of the resident hosts (Dermond *et al*., 2018, 2019*a*, 2019*b*). Hence, environmental conditions that affect the hosts directly or indirectly will have a cascading effect on the prevalence and abundance of parasites, and on the diversity of parasites infecting them (MacKenzie *et al*., 1995; Marcogliese and Cone, 1997).

In this study, we use brown trout (*Salmo trutta*, L.) and its parasite community as a model system. Adult brown trout can have migratory or sedentary habits and the occurrence of these behaviours varies among populations (Klemetsen *et al*., 2003). However, young non-migratory brown trout populations should harbour a parasite community representative of the local parasite fauna available in the stream. Fish from streams situated in geographical proximity to each other (flowing into the same lake) must be exposed to a similar regional pool of parasites in the lake available for recolonization of the streams with returning migratory adult brown trout (Barger and Esch, 2001). We hypothesize that young trout in streams with groundwater as main inflow and/or more migratory trout will harbour greater parasite diversity (species richness and composition), and more species with complex life-cycles than those in streams with mainly surface water inflow and/or less migratory trout. The aim of this study is to assess the contribution of the source of water in combination with the rate of migration of adult trout to the parasite alpha diversity and similarity in the species composition of infracommunities of young resident brown trout.

## Material and methods

### Study area and fish sampling

**Fig 1.**
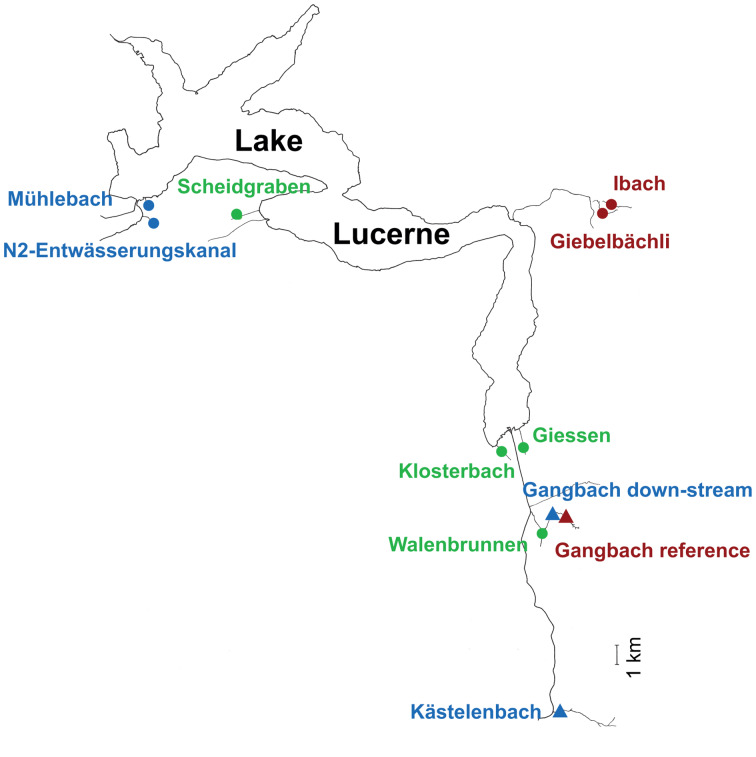
Map of Lake Luzern with the location of the 11 sampling sites situated in 10 streams. Sampling sites are colour-coded according to the category of migration rate (green, high migration; blue, medium migration; red, low or no migration) and the icons (circle, groundwater; triangle, surface water) correspond to the type of water source of the stream (see Table 1 for additional characteristics of each stream). Scale bar = 1 km.

Eleven sampling sites located in 10 streams flowing into four sections of Lake Lucerne (Switzerland) were selected on the basis of previous data on trout partial migration (J. Brodersen unpublished data) and the type of watershed (see Fig. 1 and Table 1). In this system, migratory trout generally move from the natal stream habitat to the lake as juveniles in their 3rd or 4th year of life to increase growth, and after a variable number of summers in the lake, return to their natal streams to reproduce in sympatry with lifelong resident individuals (Dermond *et al*., 2019*a*). Fish communities in the streams are dominated by brown trout, with a minority of *Cottus gobio* (L.), *Lota lota* (L.), *Esox lucius* (L.) and *Squalius cephalus* (L.) recorded in some streams. At each of the 11 sites, 26−31 juvenile brown trout were collected in October 2016 (Table 1) by electrofishing using a backpack DC unit on a survey stretch ranging from 23 to 50 m. We selected juvenile brown trout with standard length of 68–123 mm (1−2-year old) in order to ensure that the fish had never migrated and to reduce the potential effects of host age on parasite acquisition. Thus, their parasite community should reflect the locally acquired species. Fish were euthanized using a high concentration of MS222 (Tricaine Methane-Sulphonate) (Popovic *et al*., 2012; Dermond *et al*., 2019*b*). Then, all fish were weighed and measured before freezing at −20°C until parasitological examination. In addition, the trout migration rate for each stream was estimated using dual loop-antennas that recorded the transit of individually PIT-tagged fish between the lake and the streams in 2015 and 2016 as describe in Dermond *et al*. (2019*a*). Total nitrogen and phosphorus concentrations were measured from 100 mL of filtered water samples from each locality using a continuous flow analyser Skalar San++ (Skalar Analytical) and following standard methods (APHA, 1998; DEV, 2016).

**Table 1.** Characteristics of the 11 sites in the studied streams

Sites	Water source	Elevation (m)	Geographic coordinates	Migration cat.	Migration cont. (%)	Nitrogen (*μ* l^−1^)	Phosphorus (*μ* l^−1^)
Giessen	Ground	437.9	46.85179, 8.64468	High	47.3	995.7	38.3
Klosterbach	Ground	423.7	46.88732, 8.60914	High	46.8	734.9	40.2
Walenbrunnen	Ground	455.0	46.89019, 8.62386	High	35.0	789.4	43.0
Scheidgraben	Ground	437.0	46.97775, 8.40375	High	31.6	1032.5	36.0
N2-Entwässerungskanal	Ground	442.0	46.96906, 8.34617	Some	20.0	1149.9	34.1
Mühlebach	Ground	434.0	46.97809, 8.33935	Some	13.2	1097.6	41.9
Ibach	Ground	505.2	47.00872, 8.65861	None	1.0	879.6	9.2
Giebelbächli	Ground	498.0	47.00426, 8.65649	None	1.0	998.3	4.9
Kärstelenbach	Surface	516.4	46.76921, 8.67049	Some	20.0^a^	462.2	53.4
Gangbach	Surface	459.8	46.85904, 8.64863	Some	22.0	574.2	56.9
Gangbach_ref	Surface	522.2	46.86203, 8.65705	None	5.0	574.2	56.9

a This migration rate was estimated based on observed amount of returning adults in the spawning period.

### Parasite collection and morphological identification

Fish were thawed and dissected under a stereomicroscope and all the organs and tissues were examined for metazoan parasites. Parasites were fixed in absolute (99%) ethanol for molecular analyses, or in 70% ethanol for whole-mounts (Cribb and Bray, 2010; Justine *et al*., 2012). All specimens used for the morphological analyses and DNA extractions are held at the collection of the Natural History Museum of Geneva (accession numbers MHNG-PLAT-137204–MHNG-PLAT-137301). Morphological identification of the parasites was initially based on Moravec (2004), and additional comparisons with the original descriptions were performed when needed.

### Genetic analyses and molecular identification of the parasites

Molecular data were obtained from a subsample of specimens (a total of 39 specimens of 10 different morphotypes) for each prospective species to confirm their morphological identification. DNA was extracted using Chelex^®^ in deionized water containing 0.1 mg mL^−1^ proteinase K. A partial fragment of the large ribosomal subunit (28S rDNA) was chosen as marker because it is broadly used to identify parasitic flatworms (Blasco-Costa *et al*., 2016) and sequences are already available for several parasites of brown trout. The following primers were used for amplification of the 28S rDNA of trematodes and acanthocephalans, U178 (5′-GCA CCC GCT GAA YTT AAG-3′) and L1642R (5′-CCA GCG CCA TCC ATT TTC A-3′) (Lockyer *et al*., 2003); and for cestodes, LSU5F (5′-TAG GTC GAC CCG CTG AAY TTA AGC-3′) and 1500R (5′-GCT ATC CTG AGG GAA ACT TCG-3′) (Littlewood *et al*., 2000; Olson *et al*., 2003). In addition, a partial fragment of the small ribosomal subunit (18S rDNA) was amplified for the nematodes since it is the most common marker used for this group (Černotíková *et al*., 2011); with the primers PhilonemaF (5′-GCC TAT AAT GGT GAA ACC GCG AAC-3′) and PhilPCRr0 (5′-CCG TT CAA GCC ACT GC ATT A-3′). Polymerase chain reaction (PCR) amplifications were performed using a total volume of 20 *μ*L containing 4 *μ*L of DNA template, 2×  MyFi™ Mix (Bioline France, France) and 4 *μ*m of each forward and reverse primers. The PCR amplification protocol for the 28S marker followed Blasco-Costa *et al*. (2009) and for the 18S followed Černotíková *et al*. (2011). After verification of single band PCR products *via* electrophoresis, amplicons were purified with a mix of exonuclease I and thermosensitive alkaline phosphatase enzymes (Werle *et al*., 1994). Purified amplicons were sent to Macrogen Europe (Amsterdam, Netherlands) for sequencing from both strands with the same PCR primers used for amplification and an additional internal primer for the 28S fragments, L1200R (5′-GCA TAG TTC ACC ATC TTT CGG-3′) (Littlewood *et al*., 2000).

Sequences were assembled and inspected for errors in Geneious ver. 8.1.9 (Kearse *et al*., 2012) and submitted to GenBank (see accession number in Supplementary Table S1). Available sequences for taxa belonging to the same family/genus/species as our presumed taxa were obtained from GenBank and aligned together with our sequences to confirm species identification or refine the preliminary identification based on the morphology. In order to root the phylogenetic trees, a sister taxon outside the family was included for each alignment. Sequence datasets (newly obtained sequences and GenBank sequences for each target family) were aligned using default parameters in MAFFT (Katoh *et al*., 2005). The resulting alignments were visually inspected and the extremes were trimmed. Phylogenetic reconstructions were used to confirm the affiliation of our sequences to a species, genus or suprageneric taxon. We conducted phylogenetic analyses under maximum likelihood (ML) and Bayesian inference (BI) criteria. The model of nucleotide evolution general time-reversible model with the gamma-distribution among site rate variation (*Γ*) was applied to all analyses. ML analyses were conducted using RAxML ver. 8 (Stamatakis, 2006). All model parameters and bootstrap nodal support values (1000 repetitions) were estimated in RAxML. BI trees were constructed using MrBayes ver. 3.2.6 (Ronquist *et al*., 2012), running two independent MCMC runs of four chains for 10 million generations and sampling tree topologies every 1000th generation. Burn-in periods were automatically set to 25 000 generations. RAxML and MrBayes analyses were carried out for each individual dataset on the public computational resource CIPRES (Miller *et al*., 2011).

### Dataset and statistical analyses

Infection descriptors including prevalence (number of infected fish with a particular parasite in percentage) and mean intensity (mean number of parasite individuals of a species per infected fish, MI) were calculated for each site (at the component community level as described by Bush *et al*., 1997). Taxa identified exclusively with the use of molecular data (a gorgoderoid gen. sp. metacercaria, *Neoechinorhynchus* sp. and *Echinorhynchus* sp., *Streptocara incognita* and a Cystidicolidea gen. sp.) were poorly represented in the parasite communities. Thus, no specimens were available for further morphological examination. Since the remaining not sequenced specimens could not be distinguished accurately to species/genus level, the ecological parameters (prevalence and mean intensity) were calculated for the nine taxonomic entities recognized on the basis of the morphology, the three acanthocephalan lineages were pooled together and the two nematodes from the digestive tract were also grouped (see results below). Statistical analyses were carried out on the parasite infracommunities of 337 fish specimens. The dataset included information on fish migration rates, water source of the stream and water properties (total phosphorus and nitrogen concentrations) for each stream site, in addition to the parasite species and their abundance in each fish. Fish migration rates correspond to outmigration of juvenile brown trout. It was calculated as the percentage of the mean number of tagged fish leaving the stream out of the total number of tagged fish per stream over 2 years (data originally collected in 2015 and 2016 by Dermond *et al*., 2019*a*). We expect that return migration rates are linearly related to outmigration percentages except for the streams classified as non-migration, where returning migration rates are zero due to the presence of a physical barrier that impedes upstream movement.

We estimated parasite alpha diversity at the sampling unit (here the infracommunity *sensus* Bush *et al*., 1997) using three different indices. Species richness (*S*) measured the number of different taxa present in the sample. Margalef's index *D*_Mg_ from Clifford and Stephenson (1975) is used as a simple measure of diversity that only takes into account the richness and total number of individuals in the sample. Jost-transformed Shannon entropy index is used as an advanced diversity index that includes the species richness, abundance and the evenness (it weights the taxa by their frequency and treats equally common and rare species) (Jost, 2006). Differences in species richness and diversity across types of streams, migration regimes (as continuous variable) and nitrogen and phosphorus concentrations were analysed using generalized linear models (lmer4::glmer, ver. 1.1-21; Bates *et al*. (2019)) with a Poisson distribution and a negative binomial distribution (lmer4::glmer.nb), respectively, with stream as random factor.

To assess variation in species composition, the dissimilarity among the parasite infracommunities as a function of stream water source and fish migration (Krebs, 1999) was assessed by means of a non-metric multidimensional scaling (NMDS) ordination method. The zero-adjusted Bray−Curtis dissimilarity measure, which is not affected by the number of null values between samples, was used to perform the NMDS analysis (Clarke *et al*., 2006). The dissimilarity matrix was calculated upon the abundance data for each parasite species in 337 fish infracommunities. To account for species absences in some infracommunities (zero-inflated data) a ‘dummy species’ was added to all communities (see Clarke *et al*., 2006) and only parasite taxa with a prevalence of at least 5% in the whole dataset were included to estimate the zero-adjusted Bray–Curtis dissimilarity measure. The significance of the predictor variables, i.e. type of stream water source, the rate of trout migration (as continuous variable) and their interaction, was tested with the Adonis function. These analyses were conducted with the package ‘vegan’ (Oksanen, 2015) and ‘MASS’ (Venables and Ripley, 2002). All analyses were performed with the statistical software R version 3.004 (www.r-project.org).

## Results

### Parasite communities

**Fig. 2.**
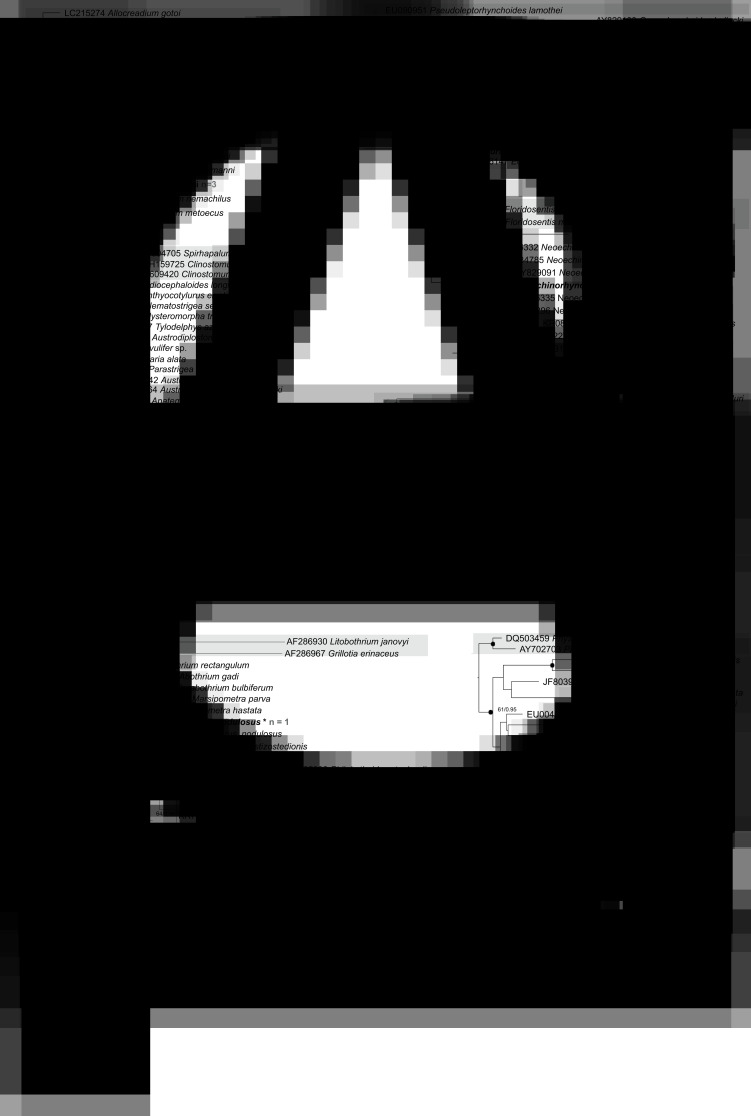
ML phylograms based on partial 28S rRNA gene sequences of parasite specimens from this study and selected sequences from GenBank: (A) *Crepidostomum*, with three sequences of taxa belonging to *Allocreadium* as outgroup; (B) the Diplostomatoidea, with sequences of *Spirhapalum* and *Clinostomum* as outgroup; (C) the Acrobothriidae, with two sequences of taxa belonging to the Gyrocotylidea as outgroup; (D) the Triaenophoridae, with two sequences of the eucestodes *Grillotia erinaceus* and *Litobothrium janovyi* as outgroup; (E) the Echinorhynchidae, with a sequence of *Pseudoleptorhynchoides lamothei* (Rhadinorhynchidae) as outgroup; (F) *Neoechinorhynchus*, with two sequences of *Floridosentis* spp. included as outgroup; (G) the Proteocephalidea, with sequences of *Gangesia parasiluri* and *Acanthobothrium* sp. serving as outgroup. ML phylograms based on 18S rRNA gene sequences of adult nematode and selected sequences from GenBank of (H) the cystidicolid nematodes, with the *Physaloptea turgida* and *P. alata* as outgroup. Bootstrap values are followed by Bayesian posterior probabilities above the branches. Full circles at the nodes illustrate high support (ML > 90, BI = 1) and empty circles illustrate moderate support (ML = 70–90, BI = 0.90–0.99). Scale-bars indicate the number of substitutions per site. Newly acquired sequences are marked in bold and 'n' indicates number of specimens sequenced. The asterisk indicates a partial sequence.

Morphological and molecular analyses of collected parasite specimens allowed the identification of a total of thirteen parasite taxa (3 allogenic and 10 autogenic) from juvenile brown trout populations in streams around Lake Lucerne. Based on the morphology alone we recognized 10 taxa: the trematodes *Crepidostomum* sp. and *Apatemon gracilis* (Rudolphi, 1819) (metacercariae); the cestodes *Cyathocephalus truncatus* (Pallas, 1781), *Proteocephalus* sp., and *Triaenophorus nodulosus* (Pallas, 1781); the monogenean *Gyrodactylus* sp.; the acanthocephalan *Echinorhynchus truttae* (Schrank, 1788); and the nematodes *Cystidicola farionis* (Fischer, 1798) and two unidentified nematodes. Molecular data and phylogenetic analyses corroborate the morphological identification of the trematodes, cestodes, *E. truttae* and *C. farionis* (see Fig. 2), and highlighted the presence of three additional taxa: an unidentified metacercaria of the Gorgodeoidea (Trematoda); *Neoechinorhynchus* sp. and *Echinorhynchus* sp. (Acanthocephala; see Fig. 2). Molecular analyses also confirmed the identification of the specimens of *Crepidostomum* sp. as *C. brinkmanni* (Faltýnková *et al.* 2020) (Fig. 2a). Sequences of juvenile specimens of *Proteocephalus* sp. formed a separate lineage among other sequences of *Proteocephalus* spp. (Fig. 2g), but did not match the sequence of *P. longicollis* available in GenBank from *Coregonus lavaretus*. Thus, the identification of these specimens to species level could not be achieved. One of the unidentified nematodes was molecularly assigned to *Streptocara incognita* (Gibson, 1968) (Acuariidae) and the other one as Cystidicolidae gen. sp.

The majority of the species in the community belong to the Platyhelminthes (present in 70% of the fish), the Acanthocephala (in 23% of the fish) and the Nematoda (in 19% of the fish; see Table 2). Most parasites were found in the digestive tract (*C. brinkmanni*, *Proteocephalus* sp., *C. truncatus*, the three acanthocephalans and the two nematodes) and only few taxa/specimens were found in other organs like the skin (*Gyrodactylus* sp.), swim bladder (*C. farionis*), liver (e.g. *T. nodulosus*) or body cavity (*A. gracilis* and a metacercariae of the Gorgodeoidea).

**Table 2.** Infection parameters for the parasite community of brown trout populations from streams around the Lake Luzern, Switzerland expressed as prevalence of infection (PI) and the mean intensity of infection (MI)

Sites	GIN		KLO		WAL		SGN		N2		MUH		IBACH		GIEB		GAN		KAR		GAN_REF	
Water source	Ground		Ground		Ground		Ground		Ground		Ground		Ground		Ground		Surface		Surface		Surface	
Migration rates (%)	47.30		46.75		35.00		31.55		20.00		13.15		1.00		1.00		22.00		20.00^a^		5.00	
	PI (%)	MI	PI (%)	MI	PI (%)	MI	PI (%)	MI	PI (%)	MI	PI (%)	MI	PI (%)	MI	PI (%)	MI	PI (%)	MI	PI (%)	MI	PI (%)	MI
*Gyrodactylus* sp.	–	–	80	4.17	–	–	–	–	67	7.10	77	4.57	77	4.26	77	6.48	10	4.67	–	–	10	4.00
*Crepidostomum brinkmanni*	100	10.90	97	41.83	97	13.00	90	5.81	90	4.04	77	2.17	13	2.25	–	–	–	–	4	1.00	–	–
Metacercariae^b^	–	–	23	1.43	–	–	–	–	23	1.42	3	1.00	–	–	3	1.00	–	–	–	–	–	–
*Cyathocephalus*	40	1.33	33	1.20	3	1.00	17	1.60	–	–	3	1.00	–	–	–	–	–	–	–	–	–	–
*truncatus*																						
*Proteocephalus* sp.	–	–	–	–	6.45	3.50	–	–	–	–	–	–	–	–	–	–	–	–	–	–	–	–
*Triaenophorus nodulosus*	–	–	–	–	–	–	–	–	–	–	7	1.50	–	–	–	–	–	–	–	–	–	–
Acanthocephala^c^:	30	2.56	57	2.59	3	1	43	1.38	–	–	37	1.36	–	–	77	4.35	–	–	–	–	–	–
*Cystidicola farionis*	20	1.17	43	2.15	–	–	3	2.00	13	1.25	–	–	–	–	13	3.00	–	–	–	–	–	–
Other nematodes^d^	27	1.88	10	1.00	–	–	3	1.00	–	–	3	3.00	20	2.00	60	6.27	–	–	–	–	–	–

WAL, Walenbrunnen, GIN, Giessen, KAR, Kärstelenbach, SGN, Scheidgraben, GAN, Gangbach down-stream, GAN_REF, Gangbach reference, KLO, Klosterbach, N2, N2-Entwässerungskanal, MUH, Mühlebach, IBACH, Ibach and GIEB, Giebelbächli.

a The migration rate in Kärstelenbach stream was not measured with an antenna. However, we assume that it goes in the category ‘some migration’. Indeed, this site is located above a barrier, which is only passed by some returning individuals and showed a low amount of lake trout spawning pits support this.

b *Apatemon gracilis* and an unidentified metacercaria of Gorgoderoidea, distinguishable only using molecular data, were analysed together.

c This group included three distinct taxa (*Echinorhynchus truttae*, *Echinorhynchus* sp. and *Neoechinorhynchus* sp.). The most common amongst the sequenced specimens was *E*. *truttae* and the two other taxa were only detected using molecular data. Thus, these three taxa were analysed together.

d This group included two distinct taxa (*Streptocara incognita* and Cystidicolidae gen. sp.). These two taxa were not common and were only distinguished using molecular data.

### Species richness and diversity

**Fig. 3.**
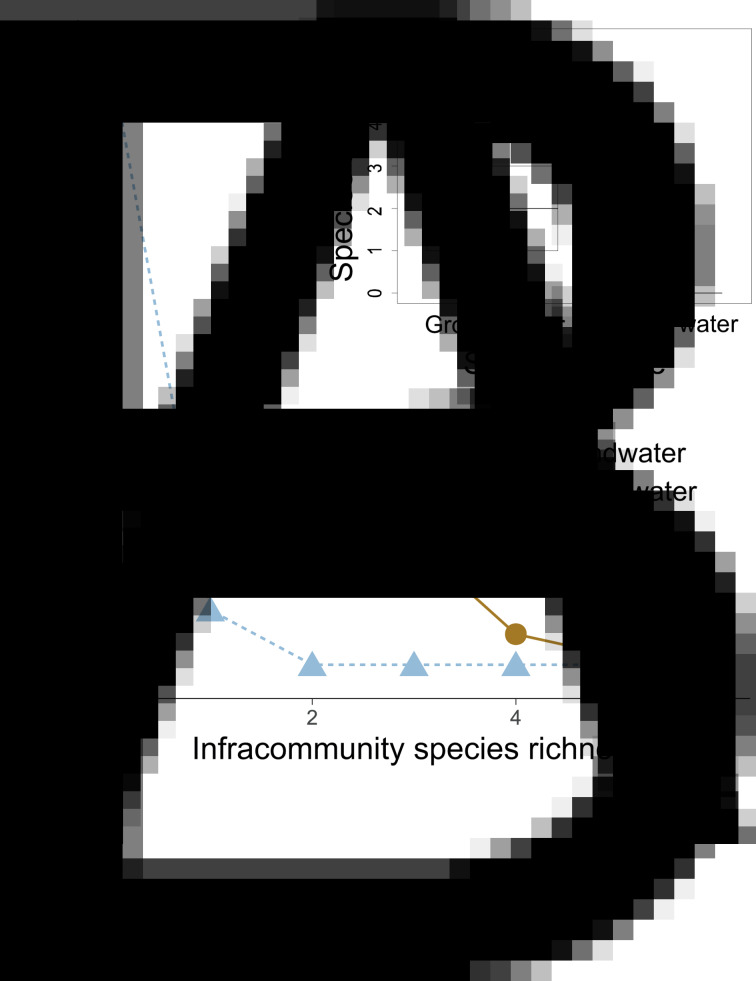
Parasite species richness in brown trout at the infracommunity level for each type of water source of the streams: (A) frequency distribution of the samples and (B) boxplot of mean species richness.

Parasite species richness was significantly different between young brown trout in groundwater-fed streams and in surface water-fed streams (*z*-value = −3.185, *P* value = 0.001; Fig. 3). Species richness at the infracommunity level ranged from 0 to 6 parasite species in groundwater streams, against 0−1 species in fish from surface water streams. No significant differences in species richness were found in relation to the rate of fish migration, nitrogen or phosphorus concentrations in the streams. Parasite species diversity (Margalef's and Shannon entropy diversity) at the infracommunity level did not show significant differences for any of the variables considered.

Out of the thirteen parasite taxa detected molecularly, only two taxa (*C. brinkmanni* and *Gyrodactylus* sp.) were shared amongst brown trout populations from both groundwater- and surface water-fed streams (Table 2). Three cestodes (*C. truncatus, T. nodulosus, and Proteocephalus* sp.), two digeneans (*A. gracilis* and the metacercaria Gorgoderoidea gen. sp.), two nematodes (*C. farionis* and an unidentified species, Nematoda gen. sp.) and three acanthocephalans (*E. truttae, Echinorhynchus sp. and Neoechinorhynchus* sp.) were only found in groundwater streams, whereas surface water streams were only colonized by *Gyrodactylus* sp. and *C. brinkmanni* (a single occurrence). Thus, almost all parasite species with complex life-cycles occurred in groundwater streams, regardless of the migration rate.

### Parasite infracommunity dissimilarities among stream types

**Fig. 4.**
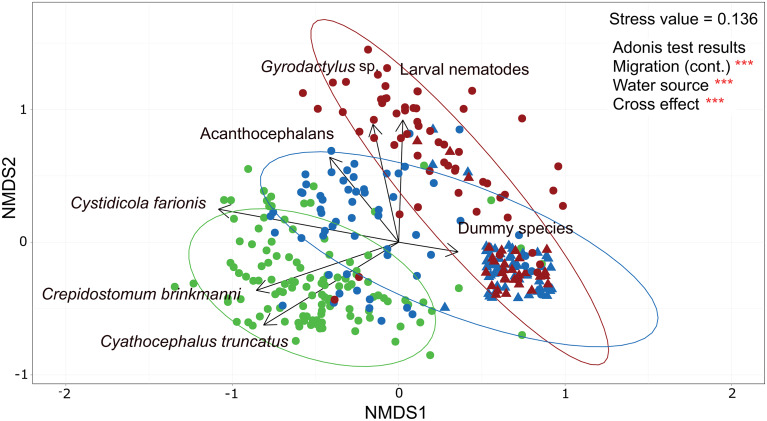
Non-metrical multidimensional scaling biplot based on Bray–Curtis dissimilarity among parasite infracommunities (*N* = 327). Infracommunities are colour-coded according to the migration category (green, high migration; blue, medium migration; red, low or no migration) and the icons (circle for groundwater; triangle for surface water) correspond to the type of water source of the stream. Ellipses regrouped 95% the parasite infracommunities of a particular migration rate category (coloured accordingly), indistinctly of the type of water source. The vectors with arrows in black indicate the contribution of each parasite taxa to the dissimilarity. Random jitter (0.1) was added to the plot to improve visualization of overlapped data points (mostly surface water infracommunities with no parasites). Asterisks represent *P* values lower than 0.001 for the Adonis test results.

Nonmetric multidimensional scaling analysis based on zero-adjusted Bray–Curtis dissimilarity metrics showed segregation in the parasite communities of brown trout between both surface water- and groundwater-fed streams and rates of migrations, despite some overlap. The analysis had a stress value of 0.136, which fell within the accepted range (<0.2; Clarke *et al*. (2006)). Both factors separately, migration rate and water source, were significant (Fig. 4, Adonis: migration rate *F*-value = 115.700, *R*^2^ = 0.211 and *P* value <0.001; water source *F*-value = 104.740, *R*^2^ = 0.191 and *P* value <0.001). The interaction between migration rate and water source was the main driver of the dissimilarity amongst the fish parasite infracommunities (Fig. 4; Adonis: *F*-value = 5.880, *R*^2^ = 0.011, *P* value <0.001). These results were consistent irrespective of whether we considered migration rate as a continuous or categorical variable.

Whereas most of the parasite infracommunities of brown trout from surface water-fed streams were aggregated on the bottom right side of the plot and characterized by the absence of parasites (i.e. ‘dummy species’ used for zero-inflated data), parasite infracommunities of brown trout from groundwater-fed streams appeared broadly spread out through the area of the biplot. Additional sub-clustering observed among the parasite infracommunities of groundwater streams (circles in Fig. 4) was captured by the variation in fish migration rates between streams. Thus, the graphical representation of the NMDS analyses and the Adonis results support heterogeneity in the parasite species composition and their abundance in the infracommunities as a function of both the source of water in the stream and the rate of returning conspecific migratory hosts from the lake. Vectors in the plot indicate that parasite infracommunities of fish from streams with high migration rates in groundwater-fed streams were dominated mainly by the presence of *C. brinkmanni*, *C. truncatus* and *C. farionis* (Fig. 4). Parasite infracommunities of trout in groundwater-fed streams with medium migration rates were mainly characterized by the presence of *C. farionis* and acanthocephalans as well as some *C. brinkmanni* and *C. truncatus*. In contrast, the parasite infracommunity composition of fish from groundwater-fed streams with low- or no migration was almost exclusively dominated by *Gyrodactylus* sp. and larval nematodes or by the absence of parasites.

## Discussion

Overall our results showed that parasite infracommunities of young brown trout inhabiting groundwater-fed streams had higher species richness than those of trout from surface water-fed streams. Whereas at a finer scale, the interaction of both the source of water and conspecific migration rates explained the dissimilarities between parasite infracommunities composition of trout individuals from different streams. Thus, both environmental stream characteristics and conspecific migration rate determine the species composition and similarity of the parasite infracommunities of resident juvenile trout.

Most of the 13 parasite taxa present in brown trout were found at an adult stage (with the exception of *T. nodulosus*, *A. gracilis* and the unidentified gordoderoid metacercaria), which suggests that brown trout do not often serve as a vehicle for transmission of parasites to other predatory fish, birds or mammals from an early age. The majority of the taxa found in this study are common parasites of European trout (e.g. Joyeux and Baer, 1936; Moravec, 2004) and already recorded in Switzerland (e.g. Dezfuli *et al*., 2002; Hahn *et al*., 2015). However, *C*. *brinkmanni* represents a new parasite record for brown trout in continental Europe. So far, only *Crepidostomum metoecus* (Braun, 1900) and *Crepidostomum farionis* (Müller, 1780) were known to infect European salmonids. *Crepidostomum brinkmanni* has been recently described from brown trout and Arctic charr in Iceland (Faltýnková *et al*., 2020) and was previously reported from mayfly in Norway (Soldánová *et al*., 2017). In addition, *Echinorhynchus* sp. may represent a putative new species or a different species yet to be described in salmonids. Indeed, *Echinorhynchus* sp. was found to differ from *E. truttae* (also found in this study) and *E. salmonis* (Müller, 1784) (previously recorded in European trout; Bauer and Skryabina (1987), Golvan (1969) and Moravec (2004)). In addition, the unidentified metacercaria belonging to the superfamily Gorgoderoidea and *Streptocara incognita* represent two new record for brown trout. The molecular characterization of specimens in addition to morphological identification in this ecological study allows us to recognize previously unknown diversity and improve the knowledge on host−parasite associations and distributions. Although we were unable to account quantitatively for these taxa in the ecological analysis, we are now aware of their presence. Our findings also highlight the need to continue to perform integrative taxonomic studies even in supposedly well-studied hosts and regions, like brown trout in central Europe.

Most of the above species were only occurring in young brown trout from groundwater-fed streams, resulting in significantly higher species richness in those streams than in surface water-fed streams. The latter were characterized by the absence of parasite infections or the presence of *Gyrodactylus* sp., a parasite with a monoxenous life-cycle, that is transmitted by contact or proximity between the hosts and whose transmission is favoured by high host densities. In our analyses, we were unable to control for host density as a determinant of the presence of this species in the different sites. The type of water source of a stream encapsulates large variation in water chemistry and nutrient availability, as well as other physical and geomorphological parameters that influence the aquatic systems and their animal communities (Ward, 1994). In surface water streams, macroinvertebrate communities are depauperated and have low abundances (Ilg *et al*., 2001). Indeed, surface water-fed streams in the area are almost devoid of snails, gammarids and coleopteran, but relatively dominated by *Ephemeroptera* (J. Brodersen unpublished data). Thus, fish may feed intensively on only a few taxa (e.g. Mejia *et al*., 2016), leading to low parasite richness and diversity. In contrast, fish have access to diverse macroinvertebrate prey in groundwater-fed streams (e.g. Kownacka and Kownacki, 1972; Ward, 1994), facilitating the acquisition of trophically transmitted parasites present in these intermediate hosts (e.g. Kennedy *et al*., 1986; Knudsen *et al*., 2007). Our results support that parasite communities in young trout from groundwater-fed streams are rich in species with complex life-cycles. Conversely, diversity estimates (using Margalef's index and the Shannon entropy index) at the infracommunity level failed to differ across different stream types. This result is likely due to the aggregated nature of the parasite infections, with most hosts having low infections and only a few carrying high values of diversity, which may require larger sample sizes in order to detect differences. Previous literature attributes this phenomenon to the low occurrences of parasite species in the infracommunities (e.g. Kennedy, 1990 and references therein).

Prior knowledge on parasite infracommunities of freshwater fish (mostly focused on lakes) suggests that parasite infracommunities are generally stochastic and unpredictable in nature, and represent an independent assortment of helminth species present in the locality (e.g. Kennedy *et al*., 1978; Esch *et al*., 1988; Kennedy, 1990). By using pre-migratory young trout, we ensured that their infracommunities reflect the parasite species available at the local scale. However, our results showed that similarity among parasite infracommunities could be predictable among streams by the interaction between water origin and the proportion of conspecific migratory individuals. This is the case at the small scale of our study, where all streams flow into the same lake. Particularly in groundwater-fed streams, where high variation in infracommunity similarity was detected, the presence of three taxa, *C. brinkmanni*, *C. truncatus* and *C. farionis* with medium to high abundances characterized streams with high migration rates of adult trout, whereas communities dominated by *C. farionis,* acanthocephalans and *Gyrodactylus* sp. or larval nematodes strengthen the similarity among parasite infracommunities of trout in streams with medium-to-low migration rates. Similarity among fish from all sites was mostly due to autogenic species, which dominated the parasite infracommunities of trout as in previous studies (e.g. Esch *et al*., 1988). Thus, we concur with Kennedy (1990) in that the major determinant of helminth assemblages in brown trout (as for eels) are the environmental and habitat conditions, in particular here the water source. Indeed, the type of water source influences the macroinvertebrate community in the stream (Ward, 1994), which in turn facilitates the transmission of parasites with complex life cycles. Furthermore, we endorse the ideas of Esch *et al*. (1990) regarding the role of host dispersal ability. Changes in the rate of migratory fish in the population can lead to changes in the similarity in composition and species abundances of the parasite assemblages at the infracommunity level of resident conspecifics. It is likely that, in the case of generalist parasites, differences caused by partial migration across populations will also affect the other non-conspecific host species in the community.

Host migration potential and colonization play an important role in structuring parasite assemblages in freshwaters (e.g. Esch, 1977; Esch *et al*., 1990; Loot *et al*., 2007; Blasco-Costa *et al*., 2013; Paterson *et al*., 2018). Whilst, differences in parasite communities have been broadly applied to distinguish fish stocks in the sea (e.g. Criscione and Blouin, 2004; Carballo *et al*., 2012; Canel *et al*., 2019), the use of metazoan parasites as biological tags in freshwater migratory fish populations has been chiefly absent (but see Criscione and Blouin, 2004). Although, geographically restricted, our results contribute to a better understanding of how variation in partial migration rates can drive the composition of parasite infracommunities of conspecific non-migrating fish in streams, and likely more broadly to other fish taxa in the case of generalist parasites. We hope future research will allow establishing the generality of the patterns detected here by investigating other partial migratory freshwater fish, across watersheds and larger scales. In addition, this knowledge can be used to inform managers about the health status of already threaten brown trout stocks, and design adequate measures to prevent parasite disease outbreaks in their natural and restocked populations.

## Supplementary material

For supplementary material accompanying this paper visit https://doi.org/10.1017/S0031182021000780. click here to view supplementary material
