# Supplementary material for: Conspecific migration and environmental setting determine parasite infracommunities of non-migratory individual fish
Source: Parasitology. 2021 May 24;148(9):1057–66. doi: 10.1017/S0031182021000780 (PMC8273861; doi:10.1017/S0031182021000780)
Supplement: Supplementary file 1 [file S0031182021000780sup.zip › S0031182021000780sup001.pdf]

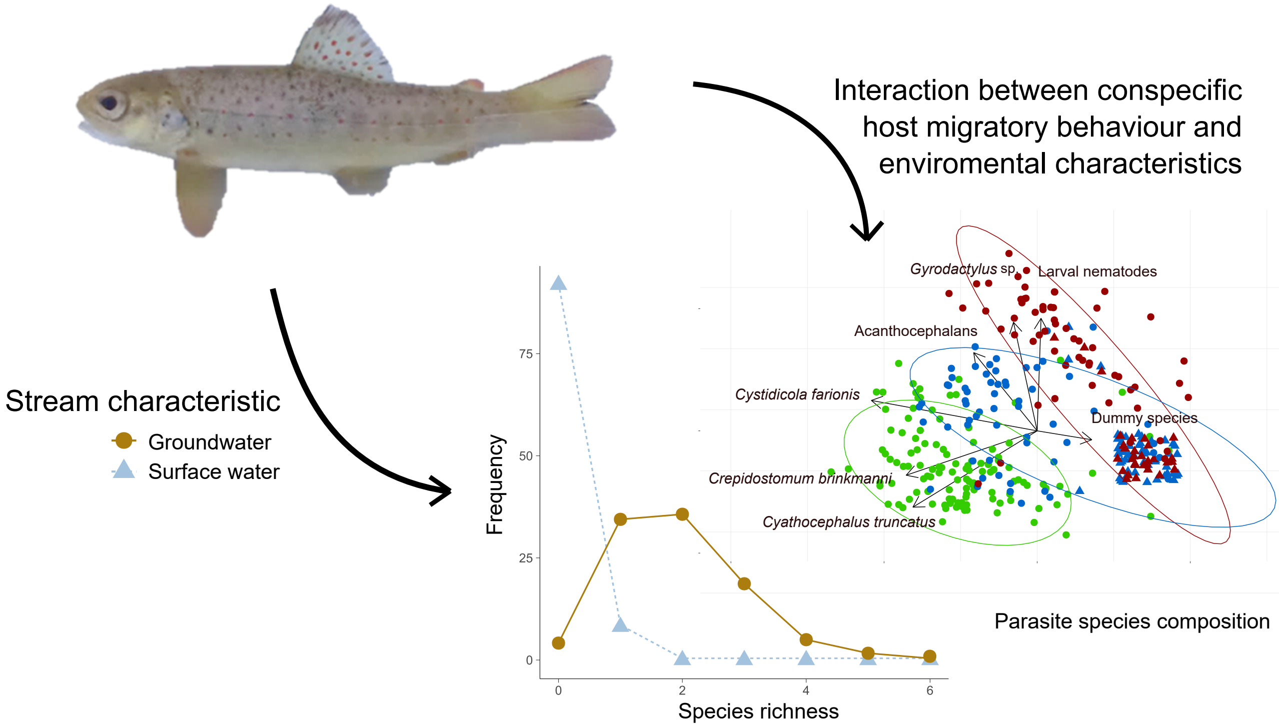

Interaction between conspecific host migratory behaviour and enviromental characteristics
Stream characteristic
Groundwater
Surface water
Frequency
Species richness
Gyrodactylus sp.
Larval nematodes
Acanthocephalans
Cystidicola farionis
Dummy species
Crepidostomum brinkmanni
Cyathocephalus truncatus
Parasite species composition
