# Supplementary material for: Conspecific migration and environmental setting determine parasite infracommunities of non-migratory individual fish
Source: Parasitology. 2021 May 24;148(9):1057–66. doi: 10.1017/S0031182021000780 (PMC8273861; doi:10.1017/S0031182021000780)
Supplement: Supplementary file 1 [file S0031182021000780sup.zip › S0031182021000780sup002.docx]

**Supplementary documentation**

**Supplementary Table S1**: Taxa included in the molecular phylogenetic analyses, their host, localities and GenBank accession numbers. Taxa in bold have been newly sequence for this study and taxa highlighted in grey have been use for rooting their respecting tree.

| **Species** | **Location** | **Host** | **GenBank ID** |
| --- | --- | --- | --- |
| **CESTODA - Acrobothriidae** | | | |
| ***Cyathocephalus truncatus*** | **Switzerland: Seedorf (Klosterbach stream)** | ***Salmo trutta*** | **MT738713** |
| ***Cyathocephalus truncatus*** | **Switzerland: Schattdorf (Giessen stream)** | ***Salmo trutta*** | **MT738714** |
| *Cyathocephalus truncatus* | Switzerland | *Salmo trutta* | AF286948 |
| *Didymobothrium rudolphii* | North Portuguese coast | *Solea lascaris* | EF095255 |
| *Spathebothrium simplex* | USA | *Liparis atlanticus* | AF286949 |
| *Diplocotyle olrikii* | UK: Scotland | *Gammarus sp.* | EF042965 |
| *Breviscolex orientalis* | Japan | *Hemibarbus barbus* | AF286910 |
| *Caryophyllaeus laticeps* | Switzerland | *Rutilus rutilus* | AF286911 |
| *Gyrocotyle urna* | Norway | *Chimaera monstrosa* | AF286924 |
| *Gyrocotyle rugosa* | USA: Alaska | *Hydrolagus colliei* | AF286925 |
| **CESTODA - Triaenophoridae** | | | |
| ***Triaenophorus nodulosus*** | **Switzerland: Stansstad (Muhlebach stream)** | ***Salmo trutta*** | **MT738716** |
| *Triaenophorus nodulosus* | UK: Scotland | *Esox lucius* | KR780879 |
| *Triaenophorus crassus* | Germany | *Coregonus lavaretus* | DQ925334 |
| *Triaenophorus stizostedionis* | USA | *Sander vitreus* | KR780900 |
| *Marsipometra hastata* | USA | *Polyodon spathula* | AY584867 |
| *Marsipometra parva* | USA | *Polyodon spathula* | KR780909 |
| *Parabothrium bulbiferum* | Norway | *Pollachius pollachius* | KR780915 |
| *Abothrium gadi* | UK | *Gadus morhua* | AF286945 |
| *Bathybothrium rectangulum* | Czech Republic | *Barbus barbus* | DQ925321 |
| *Eubothrium salvelini* | UK: Scotland | *Salvelinus alpinus* | KR780916 |
| *Eubothrium rugosum* | Russia | *Lota lota* | KR780914 |
| *Eubothrium crassum* | United Kingdom: Scotland | *Salmo salar* | KR780880 |
| *Eubothrium fragile* | United Kingdom: England | *Alosa fallax* | KR780899 |
| *Philobythoides stunkardi* | Atlantic Ocean: North Atlantic | *Alepocephalus rostratus* | DQ925332 |
| *Litobothrium janovyi* | Mexico | *Alopias superciliosus* | AF286930 |
| *Grillotia erinaceus* | United Kingdom | *Raja radiata* | AF286967 |
| **CESTODA - Proteocephalidae** | | | |
| ***Proteocephalus* sp.** | **Switzerland: Altdorf (Walenbrunnen stream)** | ***Salmo truttta*** | **MT738715** |
| *Proteocephalus macrocephalus* | River Thames, Windsor, UK | *Anguilla Anguilla* | EF095261 |
| *Proteocephalus tetrastomus* | Japan: Lake Suwa, Nagano Prefecture | *Hypomeusus nipponensis* | AJ388635 |
| *Proteocephalus longicollis* | Germany: Lake Constance | *Coregonus lavaretus* | JQ639165 |
| *Proteocephalus exiguus* | Switzerland: Lake of Bienne | *Coregonus sp.* | AJ388626 |
| *Proteocephalus percae* | Switzerland: Lake of Neuchatel | *Perca fluviatilis* | AJ388594 |
| *Proteocephalus plecoglossi* | Japan: Lake Biwa, Shiga | *Plecoglossus altivelis* | KX768939 |
| *Proteocephalus gobiorum* | Ukraine: Lake Kitay | *Neogobius fluviatilis* | KP729393 |
| *Proteocephalus demshini* | Russia: River Komissarovka, near Kamen-Rybolov | *Barbatula toni* | KX768942 |
| *Proteocephalus sagittus* | Czech Republic: Drevnice River, Vyskov, South Moravia | *Barbatula barbatula* | KP729391 |
| *Proteocephalus misgurni* | Russia: Ilistaya River, Chernigovka, Khanka Lake | *Misgurnus anguillicaudatus* | KX768941 |
| *Proteocephalus pinguis* | USA: Connecticut, Weth. Cove | *Esox lucius* | KP729395 |
| *Proteocephalus fluviatilis* | Japan | *Micropterus dolomieu* | KP729390 |
| *Proteocephalus filicollis* | UK: Scotland, Stirling | *Gasterosteus aculeatus* | AJ388636 |
| *Gangesia* *parasiluri* | Japan | *Silurus asotus* | AF286935 |
| *Acanthobothrium* sp. | Mexico | *Dasyatis longus* | AF286953 |
| **TREMATODA - Allocreadiidae** | | | |
| ***Crepidostomum brinkmanni*** | **Switzerland: Seedorf (Klosterbach stream)** | ***Salmo trutta*** | **MT738705** |
| ***Crepidostomum brinkmanni*** | **Switzerland: Stans (N2-Entwasserungskanal stream)** | ***Salmo trutta*** | **MT738706** |
| ***Crepidostomum brinkmanni*** | **Switzerland: Stansstad (Muhlebach stream)** | ***Salmo trutta*** | **MT738707** |
| *Crepidostomum brinkmanni* | Iceland: Lake Hafravatn | *Salmo trutta* | MT080773 |
| *Crepidostomum metoecus* | Russia, River near Nikolaevsk- na-Amure city | *Salvelinus leucomaensis* | FR821405 |
| *Crepidostomum nemachilus* | Russia, River near Nikolaevsk- na-Amure city | *Barbatula toni* | FR821408 |
| *Crepidostomum oschmarini* | Russia: River Il'd, upper Volga River basin | *Cottus gobio* | MH159989 |
| *Crepidostomum auritum* | USA: Pearl River, Mississippi | *Aplodinotus grunniens* | KF356373 |
| *Crepidostomum cornutum* | USA | *Lepomis gulosus* | EF032695 |
| *Crepidostomum illinoiense* | USA: Red Lake River, Minnesota | *Hiodon alosoides* | KF356372 |
| *Crepidostomum affine* | USA: Pearl River, Mississippi | *Aplodinotus grunniens* | KF356363 |
| *Crepidostomum pseudofarionis* | Iceland: Lake Hafravatn | *Salvelinus alpinus* | MT080789 |
| *Crepidostomum farionis* | Norway: Lake Takvatn | *Sphaerium* sp. | KY513136 |
| *Crepidostomum auriculatum* | Russia, River near Nikolaevsk- na-Amure city | *Huso dauricus* | FR821397 |
| *Allocreadium lobatum* | USA | *Semotilus corporalis* | EF032693 |
| *Allocreadium neotenicum* | Norway: Lake Takvatn | *Pisidium casertanum* | MH143104 |
| *Allocreadium gotoi* | Japan: Nagano, Iiyama, Midori | *Misgurnus anguillicaudatus* | LC215274 |
| **TREMATODA - Diplostomoidea** | | | |
| ***Apatemon gracilis*** | **Switzerland: Seedorf (Klosterbach stream)** | ***Salmo trutta*** | **MT728704** |
| *Australapatemon burti* | Mexico: Chicnahuapan, Estado de Mexico | *Anas diazi* | MF398342 |
| *Australapatemon* *niewiadomski* | New Zealand | *Barbronia weberi* | KT334164 |
| *Parastrigea diovadena* | Mexico: Pijijiapan, Chiapas | *Eudocimus albus* | MF398348 |
| *Uvulifer* sp. | Mexico: Ocotes, Oaxaca | *Megaceryle alcyon* | MF398333 |
| *Austrodiplostomum ostrowskiae* | Mexico: Presa la Angostura, Chiapas | *Nannopterum brasilianus* | MF398339 |
| *Tylodelphys aztecae* | Mexico: Tlahuac, Ciudad de Mexico | *Podilymbus podiceps* | MF398337 |
| *Hysteromorpha triloba* | Mexico: Tlacotalpan, Veracruz | *Nannopterum brasilianus* | MF398336 |
| *Nematostrigea serpens* | Russia North-West | *Pandion haliaetus* | KF434762 |
| *Ichthyocotylurus erraticus* | United Kingdom: Northern Ireland | *Coregonus autumnalis* | AY222172 |
| *Cardiocephaloides longicollis* | Ukraine | *Larus ridibundus* | AY222171 |
| *Alaria alata* | Poland | *Vulpes vulpes* | AF184263 |
| *Apatemon gracilis* | Norway: Lake Takvatn | *Radix balthica* | KY513176 |
| *Apatemon* sp. "jamiesoni*"* | New Zealand | *Phalacrocorax punctatus* | KT334169 |
| *Apatemon* sp. | Norway: Lake Takvatn | *Gasterosteus aculeatus* | KY513179 |
| *Clinostomum tataxumui* | Mexico: Salsipuedes Pantanos Centla, Tabasco State | *Bagre marinus* | MH159725 |
| *Clinostomum complanatum* | Italy | *Ardea cinerea* | FJ609420 |
| *Spirhapalum polesianum* | Ukraine: Lesniki, Kyiv Region | *Emys orbicularis* | AY604705 |
| **ACANTHOCEPHALA - *Echinorhynchidae*** | | | |
| ***Echinorhynchus truttae*** | **Switzreland: Seedorf (Klosterbach stream)** | ***Salmo trutta*** | **MT738711** |
| ***Echinorhynchus truttae*** | **Switzreland: Buochs (Scheidgraben stream)** | ***Salmo trutta*** | **MT738712** |
| ***Echinorhynchus* sp.** | **Switzerland: Buochs (Scheidgraben stream)** | ***Salmo trutta*** | **MT738710** |
| *Echinorhynchus truttae* | UK: Loch Walton Burn, River Carron catchment, Scotland | *Salmo trutta* | KM656147 |
| *Echinorhynchus bothniensis* | Finland: Lake Keitele | *Osmerus eperlanus* | KM656146 |
| *Echinorhynchus gadi* | Russia: White Sea, Gulf of Kandalaksha, Chupa Inlet | *Gadus morhua* | KM656150 |
| *Echinorhynchus brayi* | Atlantic Ocean: Porcupine Seabight | *Pachycara crassiceps* | KM656151 |
| *Echinorhynchus cinctulus* | Finland: Kuopio | *Lota lota* | KM656142 |
| *Echinorhynchus salmonis* | Finland: Baltic Sea, Bothnian Bay | *Coregonus lavaretus* | KM656145 |
| *Pomphorhynchus bulbocolli* | - | *Lepomis macrochirus* | AY829096 |
| *Acanthocephalus lucii* | UK: Bleasby, Nottinghamshire | *Perca fluviatilis* | KM656148 |
| *Pseudoacanthocephalus lucidus* | Japan: Shimane, Onan | *Rana ornativentris* | LC100042 |
| *Filisoma bucerium* | Middle-America | *Kyphosus elegans* | AY829110 |
| *Acanthocephaloides propinquus* | Eastern Atlantic and Mediterranean Sea | *Gobius bucchichii* | AY829100 |
| *Serrasentis sagittifer* | Australia | *Lethrinus laticaudis* | MF426926 |
| *Gorgorhynchoides bullocki* | Middle-America | *Eugerres plumiere* | AY829103 |
| *Pseudoleptorhynchoides lamothei* | Mexico | *Ariopsis guatemalensis* | EU090951 |
| **ACANTHOCEPHALA - *Neoechinorhynchidae*** | | | |
| ***Neoechinorhynchus* sp.** | **Switzerland: Stansstad (Muhlebach stream)** | ***Salmo trutta*** | **MT738708** |
| ***Neoechinorhynchus* sp.** | **Switzerland: Stansstad (Muhlebach stream)** | ***Salmo trutta*** | **MT738709** |
| *Neoechinorhynchus saginata* | America | *-* | AY829091 |
| *Neoechinorhynchus schmidti* | Pantanos de Centla, Tabasco, Mexico | *Trachemys cripta* | HQ634785 |
| *Neoechinorhynchus emyditoides* | Middle-America | *Trachemys scripta* | KR086332 |
| *Neoechinorhynchus roseum* | Laguna el Caimanero, Sinaloa, Mexico | *Achirus mazatlanus* | FJ388999 |
| *Neoechinorhynchus mexicoensis* | Middle-America | *Dormitator maculatus* | KR086312 |
| *Neoechinorhynchus mamesi* | Playa Grande, Costa Rica | *Dormitator maculatus* | KR086235 |
| *Neoechinorhynchus brentnickoli* | Middle-America | *Dormitator latifrons* | KR086227 |
| *Neoechinorhynchus chimalapasensis* | Rio Negro, Santa Maria Chimalapa, Oaxaca, Mexico | *Awaous banana* | KR086336 |
| *Neoechinorhynchus golvani* | Middle-America | *Thorichthys meeki* | KR086296 |
| *Neoechinorhynchus panucensis* | Middle-America | *Herichthys* sp. | KR086335 |
| *Floridosentis pacifica* | Mexico: Laguna de Tres Palos | *Mugil curema* | JQ436533 |
| *Floridosentis mugilis* | Middle-America | *Mugil cephalus* | AY829111 |
| **NEMATODA** | | | |
| ***Cysidicola farionis*** | **Switzerland: Seedorf (Klosterbach stream)** | ***Salmo trutta*** | **MT735337** |
| *Cystidicola farionis* | Italy | *Salmo trutta fario x Salmo marmorata* | JF803919 |
| *Ascarophis arctica* | / | Marine fish | DQ094172 |
| *Neoascarophis longispicula* | Atlantic Ocean | *Coryphaenoides mediterraneus* | JF803921 |
| *Neoascarophis macrouri* | Greenland | *Macrourus berglax* | DQ442660 |
| *Synhimantus laticeps* | Germany | *Accipiter nisus* | EU004818 |
| *Echinuria borealis* | Canada: Belcher Islands | *Somateria mollissima* | EF180064 |
| *Ascarophis adioryx* | New Caledonia | *Sargocentron spiniferum* | JF803930 |
| *Rhabdochona denudata* | Czech Republic | *Leuciscus cephalus* | DQ442659 |
| *Rhabdochona hellichi turkestanica* | India | *Schizothorax* sp. | JF803937 |
| *Rhabdochona hellichi hellichi* | Czech Republic | *Barbus barbus* | JF803913 |
| *Rhabdochona hospeti* | India | *Tor* sp. | JF803938 |
| *Rhabdochona mazeedi* | India | *Clupisoma garua* | JF803936 |
| *Salmonema ephemeridarum* | Czech Republic | *Ephemera danica* | JF803927 |
| *Spinitectus tabascoensis* | Mexico | *Ictalurus meridionalis* | JF803922 |
| *Spinitectus carolini* | USA: Belews Lake, Winston-Salem, North Carolina | *Lepomis macrochirus* | DQ503464 |
| *Metabronema magnum* | New Caledonia | *Gnathanodon speciosus* | JF803918 |
| *Heliconema longissimum* | Madagascar | *Anguilla* sp. | JF803949 |
| *Heliconema longissimum* | Japan | *Anguilla japonica* | JF803926 |
| *Turgida torresi* | Costa Rica | *Dasyprocta punctata* | EF180069 |
| *Turgida turgida* | USA: Hammond, Louisiana | *Didelphis virginiana* | DQ503459 |
| *Physasloptera alata* | Germany | Bird of prey | AY702703 |
| *Physasloptera* sp. | USA: Ohio, Franklin County | *Mephitis mephitis* | EF180065 |
| *Brugia malayi* | *India* | *Procyon lotor* | AF036588 |
| *Dirofilaria immitis* | *India* | *Canis familiaris* | AF036638 |
| *Acanthcheilonema viteae* | *India* | *Meriones unguiculatus* | DQ094171 |
| *Loa loa* | *India* | *Homo sapiens* | DQ094173 |
| *Onchoceridea* sp. | Belgium: Knokke | “Free living” stage | DQ103704 |
| *Tetrameres fissipina* | Canada: Canadian Arctic | *Somateria* spp. | EF180077 |
| *Thelazia lacrymalis* | USA: Lexington, Kentucky | *Equus caballus* | DQ503458 |
| *Serratospiculum tendo* | Germany | *Falco peregrinus* | AY702704 |
| *Spirocerca lupi* | St. Kitts | *Canis familiaris* | AY751497 |
| *Cyrnea mansioni* | Germany | Bird of prey | AY702701 |
| *Philonema oncorhynchi* | Canada | *Oncorhynchus kisutch* | DQ442670 |
| *Camallanus cotti* | / | *Lentipes concolo* | EF180071 |
